# Artificial Intelligence in Mental Health Care: Task-Specific Perspectives of Professionals in Saudi Arabia

**DOI:** 10.3390/healthcare14060701

**Published:** 2026-03-10

**Authors:** Zaenb Alsalman

**Affiliations:** Department of Family and Community Medicine, College of Medicine, King Faisal University, Al-Ahsa 31982, Saudi Arabia; zalsalman@kfu.edu.sa

**Keywords:** artificial intelligence, mental health care, digital health, psychiatry, family medicine

## Abstract

**Background**: Artificial intelligence (AI) is increasingly integrated into healthcare systems worldwide, including mental health services. While AI holds promise for improving efficiency and addressing workforce shortages, its role in psychiatry remains complex due to the central importance of empathy, clinical judgment, and ethical responsibility. Understanding clinicians’ perceptions is essential for guiding responsible AI implementation, particularly in culturally specific settings such as Saudi Arabia. **Material and Methods**: A cross-sectional survey was conducted among psychiatrists and family medicine physicians in Saudi Arabia between October and December 2025. The survey questionnaire was adapted from previously published instruments to assess perceptions of AI’s impact on mental health professions, the likelihood that AI could fully replace clinicians in ten core psychiatric tasks, expected timelines for replacement, and views on the balance between AI’s benefits and risks. Descriptive statistics, subgroup comparisons, and multivariable linear regression were used to analyze factors associated with higher perceived AI replacement likelihood. **Results**: A total of 100 physicians participated (mean age, 43.3 ± 8.9 years; 47% female). Most respondents anticipated that AI would lead to slight (45.0%) or substantial (43.0%) changes in professional roles. Perceptions varied by task: administrative tasks were most replaceable (clinical documentation, 4.03 ± 0.95; 79% likely), diagnostic/assessment tasks showed mixed perceptions (40–58%), high-risk diagnostics (suicidal/homicidal thoughts) were largely resistant (2.73–2.82; 8–30%), and relational tasks including empathetic care were least replaceable (24% likely). Physicians currently using AI tools reported significantly higher AI replacement likelihood scores, a finding that remained significant after adjustment. Overall, 64.0% of participants believed that the benefits of AI in mental health care outweighed its potential risks. **Conclusions**: Mental health professionals in Saudi Arabia largely view AI as a supportive tool rather than a replacement for clinicians. Clear boundaries remain around tasks requiring empathy and ethical judgment. These findings underscore the need for culturally sensitive, clinician-led, and ethically grounded AI integration strategies that strengthen, rather than undermine, the human foundations of mental health care.

## 1. Introduction

Mental health disorders represent a major and growing global public health challenge and contribute significantly to health-related burden worldwide [1]. Despite increased recognition of this burden, access to timely and effective mental health care remains limited in many settings due to stigma, workforce shortages, and unequal service distribution [1,2]. This burden is further shaped by digital environments, such as social media, which have been associated with adverse mental health outcomes and increased demand for mental health assessment and monitoring [3]. These challenges have driven growing interest in digital health solutions, including artificial intelligence (AI), as potential tools to improve efficiency, expand access to care, and support clinical decision-making [4]. Such technologies may be implemented as provider-facing tools that assist clinicians in documentation and decision support, or as patient-facing tools that directly engage individuals in monitoring and communication [5].

In healthcare, AI refers to computer systems designed to perform tasks that typically require human intelligence, such as pattern recognition and prediction analysis. Building on earlier clinical decision support systems, AI applications are increasingly used to assist clinicians by synthesizing complex clinical data to support diagnostic and treatment decisions [6]. AI has also been described as a “digital gatekeeper”, remaining embedded within human-led decision-making processes [4,7]. In several medical fields, particularly those reliant on pattern recognition such as radiology and pathology, AI systems have demonstrated performance comparable to human experts [8]. These advances have raised important questions about how AI may reshape clinical roles, including whether it could partially or fully replace certain physician tasks [7].

Psychiatry represents a particularly complex domain for AI integration. While mental health care increasingly relies on language-based data, behavioral patterns, and longitudinal monitoring areas well suited to AI psychiatric practice also depend heavily on empathy, therapeutic alliance, and contextual clinical judgment [9,10]. Consequently, the successful adoption of AI in mental health care depends not only on technical performance but also on clinicians’ perceptions, acceptance, and trust [11]. International studies suggest that psychiatrists generally expect AI to augment clinical practice rather than replace clinicians, particularly for tasks requiring empathy and nuanced decision-making [12,13,14].

In Saudi Arabia, the adoption of AI is accelerating as part of the national Vision 2030 agenda, which emphasizes digital transformation across healthcare services, including mental health care. Vision 2030 includes specific digital health goals aimed at modernizing the national healthcare system by improving the quality and accessibility of care, enhancing clinical efficiency, and integrating electronic health systems and AI-enabled tools [6]. AI is increasingly viewed as a means to enhance system performance and address workforce shortages; however, evidence suggests that implementation remains limited by the absence of clear strategies and practical frameworks for routine clinical integration. The Saudi healthcare system represents a distinct cultural context, in which mental health care is influenced by religious values, strong family involvement, social stigma, and expectations of physician accountability, all of which may shape clinicians’ acceptance and use of AI. Despite growing policy-level interest in AI, empirical evidence on mental health professionals’ perspectives in this context remains limited [6].

Previous research, such as the study by Sharif et al., has explored general attitudes, preparedness, and perceived professional impact of AI among mental health professionals, but has not addressed perceptions at the level of specific clinical tasks [15]. This gap is important, as implementing AI without task-specific clinician input may increase the risk of automation bias, over-reliance on algorithmic outputs, and ethical concerns related to clinical accountability and patient safety [4,7]. Addressing this gap is therefore essential for informing policy, education, and ethical governance related to the implementation of AI in mental health services.

Accordingly, the present study examined mental health professionals’ perceptions in Saudi Arabia regarding the future role of AI by assessing: (a) the expected impact of AI on mental health professions over the next 25 years; (b) the perceived likelihood that AI could fully replace clinicians across ten core psychiatric tasks, including anticipated time frames when applicable; (c) factors independently associated with higher perceived AI replacement likelihood; (d) clinicians’ overall evaluation of the balance between potential benefits and risks of AI in mental health care.

## 2. Materials and Methods

### 2.1. Study Design, Setting, and Participants

A cross-sectional study was conducted targeting physicians involved in mental health care in Saudi Arabia, specifically psychiatrists and family medicine physicians. Family medicine physicians were included because they often serve as the first point of contact for patients with mental health concerns and perform key psychiatric tasks in primary care.

As there is no prior Saudi study examining physicians’ perceptions of the potential for AI to fully replace clinicians in performing psychiatric tasks, an initial sample size was calculated assuming a prevalence of 50%. Using a 95% confidence level, a 5% margin of error, and 80% study power, the minimum required sample size was estimated to be 384 participants using Epi Info software (version 3.4.3). To account for possible non-response, the target sample size was increased by 20%.

Data were collected between October and December 2025 using an anonymous, self-administered online questionnaire distributed via email under the supervision of the Saudi Commission of Health Specialties. No identifying information was collected to ensure participant confidentiality. Due to time constraints, restricted access to eligible physicians, and reliance on voluntary participation, a total of 100 physicians completed the survey and were included in the final analysis.

### 2.2. Survey Instrument

The survey questionnaire was adapted from previously published instruments that assess physicians’ perceptions of AI replacement in clinical practice, particularly within psychiatry, to enable comparison with the international literature [12]. The questionnaire consisted of three sections. The first section collected demographic and professional characteristics, including age, gender, specialty (psychiatry /family medicine), years of clinical experience, and current use of AI-based tools for answering clinical questions or supporting decision-making. The second section assessed physicians’ perceptions of the expected impact of AI on mental health professions over the next 25 years using a four-point ordinal scale: (1) unchanged (no influence), (2) slightly changed (minimal influence), (3) substantially changed (moderate influence), and (4) obsolete (extreme influence). This section also evaluated physicians’ perceptions of whether future AI technologies could fully replace, rather than assist, human clinicians in performing ten core psychiatric tasks: (1) clinical documentation, (2) mental status examination, (3) patient interviewing, (4) detection of homicidal thoughts, (5) detection of suicidal thoughts, (6) diagnostic synthesis, (7) treatment planning, (8) referral decisions, (9) prognostic analysis, and (10) providing empathetic care. Responses were recorded using a 6-point Likert scale, ranging from “extremely unlikely” to “extremely likely,” excluding a neutral option to encourage definitive judgments, following the approach used in the adapted survey [12]. For interpretative purposes, these tasks were grouped into categories: administrative (documentation), diagnostic/assessment (structured tasks: clinical diagnoses, mental status exams, history taking; high-risk tasks: detecting suicidal or homicidal thoughts), clinical decision-making (treatment planning, referral decisions, prognoses), and relational tasks (empathetic care). In addition, for tasks rated somewhat likely, likely, or extremely likely, participants were asked to estimate the expected timeframe for AI replacement using predefined intervals (0–4 years, 5–10 years, 11–25 years, 25–50 years, >50 years). The third section explored physicians’ overall views regarding the balance between the potential benefits and risks of AI in mental health care. Participants indicated whether they believed that the benefits of AI outweighed its potential harms (yes/no).

### 2.3. Statistical Analysis

Data were analyzed using the Statistical Package for the Social Sciences (SPSS) version 25. Continuous variables were summarized as means and standard deviations, while categorical variables were presented as frequencies and percentages. For the ten psychiatric tasks, a composite AI replacement likelihood score was calculated by averaging the 6-point Likert-scale ratings across all tasks. Although individual Likert items are ordinal in nature, they were treated as approximately interval-level variables for the calculation of mean scores and for subsequent parametric analyses (independent-sample t-tests and multivariable linear regression), consistently with common practice in survey research. For selected analyses, Likert-scale responses were dichotomized into likely (somewhat likely, likely, extremely likely) and unlikely (somewhat unlikely, unlikely, extremely unlikely). Subgroup analyses were conducted to examine differences in AI replacement perceptions by specialty (psychiatrist vs. family medicine physician), gender, years of clinical experience (<5, 5–10, and >10 years), and current use of AI tools. Independent-sample t-tests were used to compare mean AI replacement likelihood scores between groups, and chi-square tests were applied for categorical comparisons. To identify independent predictors of higher AI replacement likelihood, multivariable linear regression analysis was performed, including participant characteristics (gender, specialty, current use of AI tools, and years of professional experience). Physicians with <5 years of experience were used as the reference group to represent early-career clinicians. This analysis was exploratory in nature and aimed to identify potential associations rather than test predefined hypotheses. Regression results were reported as beta coefficients with 95% confidence intervals. All statistical tests were two-sided, and a *p*-value of <0.05 was considered statistically significant.

## 3. Results

A total of 100 physicians completed the survey. The mean age of respondents was 43.3 ± 8.9 years, and women accounted for nearly half of the sample (47%). Psychiatrists comprised 34% of participants, while the remaining 66% were family medicine physicians. Clinical experience was broadly distributed, with 9% of respondents having less than 5 years of practice, 25% reporting 5–10 years of experience, and 66% having more than 10 years of experience. The majority of physicians (69%) reported current use of AI-based tools to answer clinical questions (Table 1).

**Figure 1:**
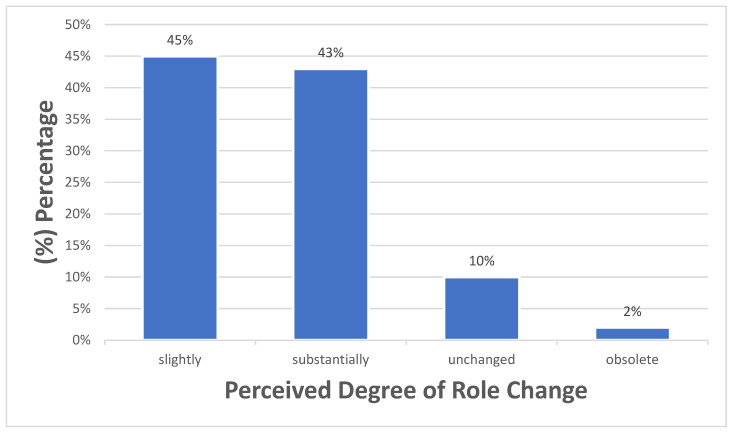
Perceived impact of Al on mental health professions (n = 100).

Regarding the future impact of AI on mental health professions, most participants anticipated some degree of change in their professional roles. Nearly half expected roles to change slightly (45.0%), while a similar proportion anticipated substantial changes (43.0%), as illustrated in Figure 1. Across the ten psychiatric tasks assessed, the overall mean AI replacement likelihood score was 3.19 ± 0.74. Perceptions varied notably by task. Administrative tasks were seen as most replaceable, with clinical documentation rated 4.03 ± 0.95 and 79% of respondents indicating likely. Diagnostic/assessment tasks showed mixed perceptions (40–58%), whereas high-risk diagnostic tasks like detecting suicidal or homicidal thoughts were rated as largely resistant to automation (means of 2.73–2.82 and 8–30%). Clinical decision-making tasks had moderate perceived replaceability, and relational tasks were the least replaceable, with empathetic care rated 2.57 ± 1.24 (24% likely) (Table 2).

Regarding subgroup analyses of the overall AI replacement likelihood score, family medicine physicians reported significantly higher AI replacement likelihood scores than psychiatrists (unadjusted *p* = 0.027), and physicians who currently used AI tools reported higher scores than non-users (unadjusted *p* = 0.002) (Table 3). In multivariable linear regression analysis, current AI use remained independently associated with higher AI replacement likelihood after adjustment for relevant covariates (B = 0.47; 95% CI: 0.153 to 0.781; *p* = 0.004) (Table 4).

**Figure 2:**
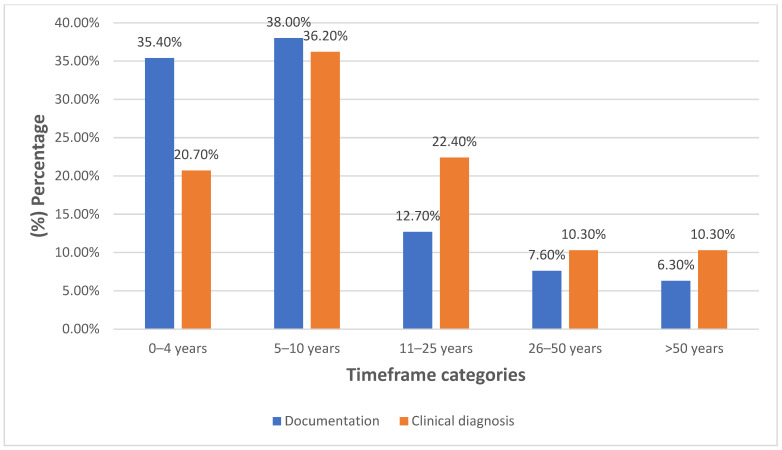
Expected timeframe for Al replacement among respondents rating tasks.

**Figure 3:**
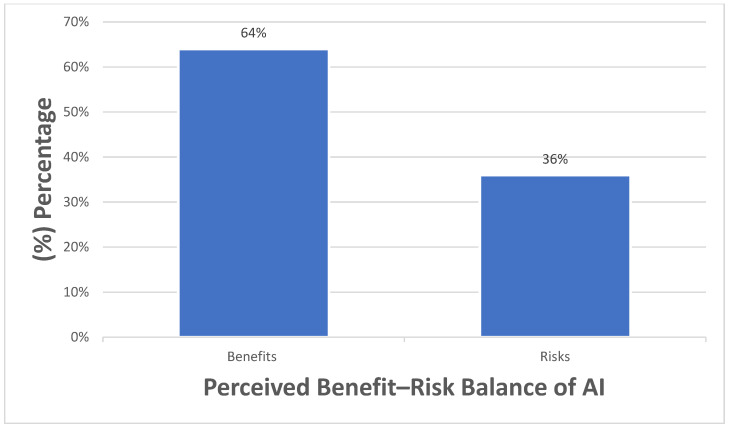
Perceived balance of benefits and risks of Al in mental health care (n = 100).

Among respondents who considered AI likely (somewhat likely, likely, extremely likely) to replace clinicians in documentation or diagnostic tasks, most anticipated that such replacement would occur within the next 10 years (Figure 2). Overall, nearly two-thirds of participants (64.0%) reported that the potential benefits of AI in mental health care outweighed its associated risks (Figure 3).

## 4. Discussion

This study provides insight into physicians’ views on the evolving role of AI in mental health care within Saudi Arabia. The findings suggest that while clinicians anticipate meaningful changes in practice, they do not expect full replacement across psychiatric care. AI was viewed as most suitable for structured and administrative tasks, whereas relational and high-risk diagnostic tasks were considered largely resistant to automation.

Consistently with previous studies, participants perceived AI primarily as a supportive tool capable of reshaping clinical workflows while preserving the physician’s central role [12,16,17]. Administrative tasks, particularly clinical documentation, were seen as the most amenable to automation, with nearly four out of five respondents rating documentation as likely or extremely likely to be replaced, many within the next 5–10 years. This aligns with prior research showing that repetitive, rule-based functions are early targets for AI integration and may reduce administrative burden and burnout [12,16,18,19]. In contrast, perceptions varied across diagnostic and relational domains. Structured diagnostic tasks (clinical diagnoses, mental status examinations, and history taking) were viewed as moderately replaceable, whereas high-risk responsibilities such as suicide or homicide risk detection were considered far less suitable for automation. Tasks requiring empathy and therapeutic alliance were rated as least replaceable. This pattern likely reflects differences in task structure and perceived clinical risk: structured diagnostic formulation typically follows standardized criteria and pattern recognition processes, making it more compatible with AI assistance, whereas high-risk tasks require contextual judgment and clear accountability. Similarly, relational tasks (empathetic care) were consistently rated as least replaceable, reinforcing the view that AI is limited in domains requiring empathy, human judgment, and therapeutic alliance [12,20]. Together, these findings suggest that clinicians draw an implicit boundary between procedural or structured tasks suitable for AI support and relational or high-risk responsibilities that require human oversight [10,17,20,21,22].

Differences between professional groups were also observed. Psychiatrists tended to be more cautious than family medicine physicians regarding AI’s potential to replace clinical tasks, although this difference was attenuated after adjustment. This may reflect psychiatrists’ greater exposure to the complexity and subjectivity inherent in mental health care. Moreover, unlike Olijo et al., our sample was nearly equal in gender, and no significant differences were observed in perceptions of AI’s ability to replace clinical tasks [23]. Notably, the current use of AI tools emerged as a key factor shaping perceptions. Physicians who already used AI for clinical questions or decision support were more likely to view AI as capable of replacing certain tasks, even after multivariable adjustment. This pattern may be explained by greater AI literacy and hands-on experience: familiarity with AI reduces uncertainty and helps clinicians recognize tasks AI can reliably support, whereas non-users may overestimate risks. Targeted education and practical exposure are therefore important to align perceptions and support informed AI integration in mental health care [12,15,16]. These findings are consistent with prior research. Sharif et al. reported more positive perceptions among clinicians familiar with AI, underscoring the importance of improving AI literacy [15]. Similarly, Dimeff et al. found that the successful adoption of AI-assisted interventions largely depends on staff acceptance and their willingness to integrate these tools into routine clinical workflows [24]. Overall, greater familiarity appears to foster more balanced and realistic expectations of AI’s supportive role in clinical care [12,25].

Despite ongoing concerns, nearly two-thirds of participants in our study felt that the benefits of AI in mental health care outweigh its potential harms. In contrast, Doraiswamy et al. reported much lower optimism, with only 36% of psychiatrists believing that AI’s future benefits would outweigh its risks [12]. This difference may be partly explained by timing, as the Doraiswamy et al. study was conducted in 2019, before the COVID-19 pandemic, when clinical exposure to AI and digital mental health tools was more limited. The rapid acceleration of telemedicine, digital health adoption, and AI-supported clinical workflows during and after the pandemic may have contributed to greater familiarity and more favorable perceptions in our sample. However, this optimism should be interpreted with caution. Prior research has consistently raised concerns about over-reliance on automated systems, accountability for errors, algorithmic bias, and potential risks to patient safety, as widely discussed in the AI ethics and healthcare literature [25,26,27,28]. These concerns mirror international ethical analyses emphasizing the dangers of automation bias, unclear accountability, and patient safety risks when AI systems are deployed without robust governance frameworks [29]. A recent review of 85 studies shows that AI-enabled tools can aid in mental health diagnosis, monitoring, and intervention; however, balancing privacy protection with support for high-risk individuals remains a key challenge [17]. Such challenges are particularly salient in non-Western settings, where AI systems trained on external or non-local datasets may inadequately capture local cultural norms, clinical practices, and patient expectations. This highlights the need for clinician-led, context-aware approaches to AI adoption to ensure relevance and ethical use [18]. In addition, the use of AI in mental health care should be guided by clear protocols and approved by ethics committees across all participating centers to ensure participant safety and privacy [30].

In Saudi Arabia, the cultural landscape of mental health care adds further complexity. Psychiatric practice is deeply shaped by religious values, strong family involvement, and expectations of personal trust and physician accountability. Patients often prefer guidance that respects Islamic counseling traditions, and sensitive topics such as suicide, marital issues, or gender-related concerns typically require culturally tailored, gender-sensitive communication, which generic global AI chatbots may not fully provide [31,32,33,34]. Vision 2030 is rapidly advancing digital health in Saudi Arabia, encouraging clinicians to integrate telehealth and AI into everyday practice [6]. Yet hesitation to entrust AI with empathetic care or suicide risk assessment reflects deeply rooted ethical and cultural values. AI tools built on non-local data therefore need careful adaptation to reduce cultural bias and ensure they are safe and appropriate for local patients.

To support AI-enhanced practice, Saudi medical education could integrate practical AI training into residency programs and continuing education, including short workshops on AI ethics, case-based exercises using decision-support tools, and foundational skills such as prompt engineering. Ongoing digital literacy training should also be emphasized to maintain competence as technologies evolve and to ensure AI interventions remain culturally appropriate and effective beyond pilot implementation. These strategies build AI literacy, ethical awareness, and practical skills among clinicians while promoting responsible and confident use of AI in clinical care [35].

This study has several strengths, including a task-specific approach that allows meaningful comparison with the international literature. Instead of viewing AI as a uniform entity, this approach mirrors real-world clinical practice, in which tasks vary widely in their complexity, clinical responsibility, and perceived risk. To our knowledge, it is the first study to examine physicians’ perceptions of AI replacement in mental health care within Saudi Arabia, offering contextually grounded insights into local clinical workflows, cultural factors, and health-system dynamics. Nevertheless, limitations should be acknowledged. The cross-sectional design captures perceptions at a single point in time, and the relatively small sample size may have limited the statistical power of the multivariable regression analysis; therefore, these findings should be interpreted as exploratory and hypothesis generating. Future studies should be longitudinal, larger, and include other professionals such as psychologists and nurses to enhance generalizability.

## 5. Conclusions

In summary, mental health providers in Saudi Arabia recognize AI as a transformative force in mental health care with substantial benefits for administrative and structured tasks. However, they maintain firm boundaries around relational and high-risk diagnostic tasks, such as empathetic care and suicide risk assessment, which are considered largely resistant to automation. These findings underscore the importance of ethically grounded, culturally sensitive, and clinician-led AI implementation strategies that strengthen rather than diminish the human foundations of mental health care.

## Figures and Tables

**Table 1:** Participant characteristics (*N* = 100).

Characteristic	Value
Age, mean ± SD (years)	43.3 ± 8.9
Gender	
Male	53 (53.0%)
Female	47 (47.0%)
Profession	
Family medicine physician	66 (66.0%)
Psychiatrist	34 (34.0%)
Years of clinical experience	
>10 years	66 (66.0%)
5–10 years	25 (25.0%)
<5 years	9 (9.0%)
Current use of AI tools	
Yes	69 (69.0%)
No	31 (31.0%)

**Table 2:** Perceived likelihood that AI could replace clinicians for specific psychiatric tasks.

Task	Mean ± SD	Likely %	Unlikely %
Update records/documentation	4.03 ± 0.95	79.0	21%
Provide empathetic care	2.57 ± 1.24	24.0	76%
Formulate personalized treatment plans	3.20 ± 1.15	48.0	52%
Decide on outpatient vs. inpatient referral	3.21 ± 1.09	45.0	55%
Establish prognoses	3.49 ± 0.94	57.0	43%
Detect acute homicidal thoughts	2.82 ± 1.07	30.0	70%
Detect suicidal ideation	2.73 ± 0.87	8.0	92%
Reach clinical diagnoses	3.52 ± 0.93	58.0	42%
Perform mental status exams	3.02 ± 1.16	40.0	60%
Conduct patient interviews/history taking	3.36 ± 1.17	53.0	47%

**Table 3:** Subgroup analysis of the overall AI replacement likelihood score.

Variable	AI Likelihood Score (Mean ± SD)	Effect	*p*-Value
Age (years)		r = 0.11	0.262
Gender: Female	3.12 ± 0.74		0.339
Gender: Male	3.26 ± 0.75		
Profession: Psychiatrist	2.97 ± 0.69		0.027
Profession: Family medicine physician	3.31 ± 0.75		
Years of experience: <5 years	3.26 ± 0.79		0.910
Years of experience: 5–10 years	3.14 ± 0.73		
Years of experience: >10 years	3.21 ± 0.75		
Use of AI tools: Yes	3.35 ± 0.72		0.002
Use of AI tools: No	2.85 ± 0.69		

**Table 4:** Multivariable linear regression predicting higher AI replacement likelihood score.

Predictor	95% CI	*p*-Value
Male (vs. female)	−0.104 to 0.469	0.209
Family medicine (vs. psychiatrist)	−0.052 to 0.566	0.102
Experience 5–10 years (vs. <5 years)	−0.784 to 0.322	0.408
Experience >10 years (vs. <5 years)	−0.867 to 0.284	0.317
Uses AI tools (yes vs. no)	0.153 to 0.781	0.004
Age (per year)	−0.003 to 0.036	0.105

## Data Availability

The data are not publicly available due to ethical restrictions and the need to protect participant confidentiality, as the dataset contains sensitive professional opinions collected from a relatively small sample of physicians.
